# Genetic and genomic monitoring with minimally invasive sampling methods

**DOI:** 10.1111/eva.12600

**Published:** 2018-03-24

**Authors:** Emma L. Carroll, Mike W. Bruford, J. Andrew DeWoody, Gregoire Leroy, Alan Strand, Lisette Waits, Jinliang Wang

**Affiliations:** ^1^ Scottish Oceans Institute and Sea Mammal Research Unit University of St Andrews St Andrews UK; ^2^ Cardiff School of Biosciences and Sustainable Places Research Institute Cardiff University Cardiff, Wales UK; ^3^ Department of Forestry and Natural Resources and Department of Biological Sciences Purdue University West Lafayette IN USA; ^4^ Animal Production and Health Division Food and Agriculture Organization of the United Nations Rome Italy; ^5^ Grice Marine Laboratory Department of Biology College of Charleston Charleston SC USA; ^6^ Department of Fish and Wildlife Sciences University of Idaho Moscow ID USA; ^7^ Institute of Zoology Zoological Society of London London UK

**Keywords:** conservation genetics, DNA fingerprinting, individual identification, noninvasive genetic sampling, population demography, wildlife forensics, wildlife management

## Abstract

The decreasing cost and increasing scope and power of emerging genomic technologies are reshaping the field of molecular ecology. However, many modern genomic approaches (e.g., RAD‐seq) require large amounts of high‐quality template DNA. This poses a problem for an active branch of conservation biology: genetic monitoring using minimally invasive sampling (MIS) methods. Without handling or even observing an animal, MIS methods (e.g., collection of hair, skin, faeces) can provide genetic information on individuals or populations. Such samples typically yield low‐quality and/or quantities of DNA, restricting the type of molecular methods that can be used. Despite this limitation, genetic monitoring using MIS is an effective tool for estimating population demographic parameters and monitoring genetic diversity in natural populations. Genetic monitoring is likely to become more important in the future as many natural populations are undergoing anthropogenically driven declines, which are unlikely to abate without intensive adaptive management efforts that often include MIS approaches. Here, we profile the expanding suite of genomic methods and platforms compatible with producing genotypes from MIS, considering factors such as development costs and error rates. We evaluate how powerful new approaches will enhance our ability to investigate questions typically answered using genetic monitoring, such as estimating abundance, genetic structure and relatedness. As the field is in a period of unusually rapid transition, we also highlight the importance of legacy data sets and recommend how to address the challenges of moving between traditional and next‐generation genetic monitoring platforms. Finally, we consider how genetic monitoring could move beyond genotypes in the future. For example, assessing microbiomes or epigenetic markers could provide a greater understanding of the relationship between individuals and their environment.

## 1 INTRODUCTION

The current era of rapid global environmental change (Zalasiewicz, Williams, Haywood, & Ellis, 2011) is predicted to lead to a rapid loss of biodiversity (Pimm et al., 2014). To assess and mitigate the impact of this loss, many national and international organizations have established biodiversity monitoring strategies (e.g., Kurtz, Jackson, & Fisher, 2001; United Nations Environment Programme Convention on Biological Diversity SBSTTA, 2003). Key tools for biodiversity monitoring utilise methodological approaches from the field of genetic monitoring, relying on genetic tools for evaluating change (Stetz, Kendall, Vojta, & GeM, 2011). Genetic monitoring focuses on quantifying temporal changes in population genetic metrics, or other population data, generated using molecular markers (Schwartz, Luikart, & Waples, 2007). Genetic monitoring can be used to estimate many biological parameters of interest, including demographic parameters such as abundance, vital rates, occupancy, hybridization, disease status; population genetic parameters including genetic diversity, structure and effective population size; and increasingly, responses to selective pressures such as exploitation (e.g., trophy hunting) and climate change (Schwartz et al., 2007; Stetz et al., 2011). Here, we examine genetic monitoring approaches that use noninvasive (e.g., naturally shed feathers) or minimally invasive (e.g., biopsy darts, buccal swabs) samples (hereafter MIS) because wildlife ecology and conservation has benefitted greatly from the new data provided by these approaches (Beja‐Pereira, Oliveira, Alves, Schwartz, & Luikart, 2009).

Genetic monitoring using MIS approaches was first introduced in 1992 as a method to obtain genetic samples from brown bears (*Ursus arctos;* Höss, Kohn, Pääbo, Knauer, & Schröder, 1992; Taberlet & Bouvet, 1992; see Box 1) and to study social structure in chimpanzees (*Pan troglodytes;* Morin & Woodruff, 1992). MIS has become the method of choice for genetic monitoring of many vertebrate species. This is because sampling of hair, faeces, remote skin biopsies or feathers provides DNA from free‐ranging animals that can be used to identify individuals across time and space and generates genetic data without having to catch, handle or in some cases, even observe them (Beja‐Pereira et al., 2009; Schwartz et al., 2007; Waits & Paetkau, 2005). In the last 25 years, researchers have demonstrated a variety of important applications of MIS including detecting rare species (Palomares, Godoy, Piriz, O'Brien, & Johnson, 2002; Valière et al., 2003), estimating population size and other demographic parameters (Carroll et al., 2013; Kendall et al., 2009; Kohn et al., 1999; Rudnick, Katzner, Bragin, Rhodes, & DeWoody, 2005; Woodruff, Lukacs, Christianson, & Waits, 2016; Woods et al., 1999), evaluating genetic diversity and gene flow (Epps et al., 2005; Gerloff, Hartung, Fruth, Hohmann, & Tautz, 1999; Lucchini et al., 2002; Palsbøll et al., 1997), detecting movement and migration (Dixon et al., 2006; Proctor, Mclellan, Strobeck, & Barclay, 2005), evaluating social structure (Constable, Ashley, Goodall, & Pusey, 2001; Ford et al., 2011; Morin et al., 1994), detecting hybridization (Adams, Kelly, & Waits, 2003; Bohling et al., 2016; Steyer et al., 2016), monitoring disease epizootics (Kohn & Wayne, 1997; Schunck, Kraft, & Truyen, 1995), identifying diet items (De Barba et al., 2014; Höss et al., 1992; Taberlet & Fumagalli, 1996) and wildlife forensic applications (Banks, Horsup, Wilton, & Taylor, 2003; Ernest, Rubin, & Boyce, 2002; Lukoshek et al., 2009; Wasser et al., 2004).

Box 1 **Brown bears (Ursus arctos) as a model system for the development of MIS approaches**

1

**Figure 1:**
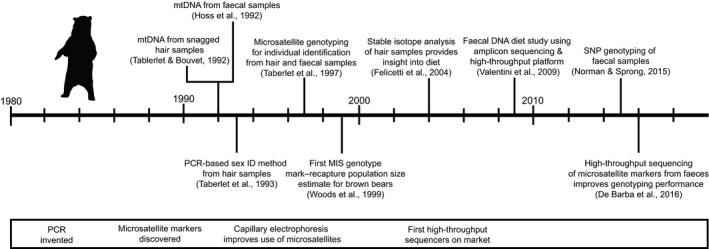

The brown bear is the most widely distributed bear species and is locally endangered at many locations across its range. The desire for alternative methods to monitor this charismatic species launched the field of noninvasive genetic sampling, and the field has kept pace with technological developments. First, Taberlet and Bouvet (1992) and Höss et al. (1992) demonstrated that mitochondrial DNA (mtDNA) sequences could be obtained from snagged hair and faecal samples, respectively. Höss et al. (1992) were also the first to demonstrate the ability to amplify diet items in scat by sequencing a 356 bp *rbcL* chloroplast sequence to identify the dominant plant in their diet (*Photinia villosa*). These were the first studies to document successful amplification of DNA from hair and faecal samples of wild species. Soon researchers were amplifying nuclear DNA to determine sex (Taberlet, Mattock, Dubois‐Paganon, & Bouvet, 1993) and for individual identification (Taberlet et al., 1997). This work was critical to the understanding of microsatellite genotyping errors and approaches for minimizing their impact on MIS data sets (Taberlet et al., 1996). MIS was then used extensively in Europe in the 1990s to obtain data on genetic diversity, genetic structure, phylogeography and minimum counts of population size (Kohn, Knauer, Stoffella, Schroder, & Paabo, 1995; Taberlet & Bouvet, 1992; Taberlet et al., 1997). In the late 1990s, North American researchers embraced MIS methods as an alternative approach for population estimation and produced the first mark–recapture population estimates using DNA extracted from brown bear hair samples collected from barbed‐wire hair snares (Mowat & Strobeck, 2000; Woods et al., 1999), which revolutionised methods for estimating population size (Boulanger, Himmer, & Swan, 2004; Kendall et al., 2009). This approach was expanded to couple stable isotope analysis of hair samples with genetic analysis to provide a new approach for noninvasively determining the number of brown bears in Yellowstone park feeding on cutthroat trout and estimating the number of fish consumed per year by bears (Felicetti et al., 2004; Haroldson et al., 2005; Teisberg et al., 2014). MIS applications have expanded to include obtaining DNA from saliva on mammalian (Farley, Talbot, Sage, Sinnott, & Coltrane, 2014) and salmonid (Wheat, Allen, Miller, Wilmers, & Levi, 2016) carcasses to conduct species and individual identification. MIS has been the main method used to track small remnant or reintroduced populations in Europe (e.g., De Barba, Waits, Garton, 2010; Karamanlidis et al., 2010), Pakistan (Bellemain, Nawaz, Valentini, Swenson, & Taberlet, 2007), western continental United States (Proctor et al., 2012; Romain‐Bondi et al., 2004) and the Gobi desert (McCarthy, Waits, & Mijiddorj, 2009; Tumendemberel et al., 2015). Brown bears have also been an important model system for the transition from genetic to genomic approaches in MIS. For example, they have been the focus of dietary metabarcoding studies (De Barba et al., 2014; Valentini et al., 2009). Recently, new approaches were developed to sequence PCR‐amplified microsatellites on an Illumina platform to obtain multilocus genotypes from brown bears (De Barba et al., 2017). This approach increased success rates by 20%–30% and decreased costs per sample by 40% compared to traditional capillary electrophoresis genotyping of microsatellite loci. Also, SNP loci have been identified for brown bears and successfully genotyped for faecal samples using the Fluidigm platform (Norman & Spong, 2015; Spitzer, Norman, Schneider, & Spong, 2016). These advancements using genomic methods provide much promise for the continued noninvasive genetic monitoring of brown bears across their range. The figure shows the timeline of the key advances in using MIS for genetic monitoring of brown bears, along with the approximate timing of some key molecular methods.

There is now a wealth of published evidence that MIS is comparable in costs or more cost‐effective (De Barba, Waits, Genovesi, et al., 2010; Solberg, Bellemain, Drageset, Taberlet, & Swenson, 2006) than traditional methods (e.g., camera trapping, tracks and signs and even trapping animals) and that collection and analysis of larger genetic sample sizes are often possible (De Barba, Waits, Garton, et al., 2010; Marucco et al., 2009; Solberg et al., 2006; Stenglein, Waits, Ausband, Zager, & Mack, 2010), prompting many wildlife managers to shift to MIS approaches. Extensive methodological and analytical development has been invested in establishing protocols to maximise success rates and minimise error rates when using these low‐quality DNA sources for genetic monitoring (Beja‐Pereira et al., 2009; Broquet & Petit, 2004; Miquel et al., 2006; Morin et al., 2010; Smith & Wang, 2014; Taberlet et al., 1996; Taberlet & Luikart, 1999; Waits & Paetkau 2005; Wang, 2016). Genetic monitoring is set to become more important in the future, largely because many vertebrate species have undergone rapid, anthropogenic population declines (Li et al., 2016) that are unlikely to abate without intensive management efforts. Fortunately, the genomic revolution of the early 2000s has given rise to a variety of more precise or more powerful molecular techniques that will make genetic monitoring even more effective in the future.

New technologies for genetic monitoring typically rely upon single nucleotide polymorphisms, or SNPs (Morin, Luikart, & Wayne, 2004). Unlike more conventional DNA markers such as microsatellites, SNPs have relatively few alleles per locus (theoretically up to four but usually only two due to low mutation rates; Glaubitz, Rhodes, & DeWoody, 2003) and often have more limited application across species than microsatellite markers, often being species‐specific. In addition, SNP loci are more prone to ascertainment bias, as they are selected because of their high polymorphism in the populations of interest but are often monomorphic in even closely related populations (Gautier et al., 2009). However, SNP‐based approaches have great potential for noninvasive genotyping as (i) large numbers of loci can be surveyed simultaneously, particularly with next‐generation sequencing or genotyping platforms, and (ii) the relative ease of scoring, analysis and modelling of SNP genotype data due to the digital/binary nature of the data. The latter point contrasts favourably with the near continuous distribution of microsatellite alleles that can be difficult to consistently characterise and thus could cause scoring errors.

For these reasons, we focus this review on recent genomic methods and platforms for producing SNP genotypes from MIS, considering factors such as development costs and error rates. We evaluate whether these new approaches will enhance our ability to investigate questions in genetic monitoring, such as estimating abundance, genetic structure and relatedness. As the field is in a period of unusually rapid transition, we also highlight the importance of legacy data sets and recommend how to address the challenges of moving between traditional and next‐generation genetic monitoring platforms. Finally, we consider how genetic monitoring could move beyond genotypes in the future. For example, assessing microbiomes could provide a greater understanding of the relationship between individuals and their environment.

## 2 SAMPLING AND METHODOLOGICAL CONSIDERATIONS

### 2.1 Sampling issues

Sampling strategies for non‐ or minimally invasive material in the natural environment depend on the research aims and objectives at hand and can be conducted randomly, opportunistically or using standardised designs. For example, sampling strategies may be designed to maximise the total number of individuals detected (typically used for minimum census estimates and population genetic studies) or to maximise recaptures using high intensity sampling over a limited geographic range (to estimate ranging behaviour or territory size for an individual or group of individuals, and to estimate population size, e.g., Rudnick, Katzner, Bragin, & DeWoody, 2008). When considering the estimation of many population genetic parameters, sampling should be designed to be random with respect to kin (this can also be addressed by *post hoc* data pruning, but see Waples & Anderson, 2017). It is also important to consider the temporal sampling interval, which can affect sample sizes, genotyping success rates, genotyping error rates and impact the ability to meet modelling assumptions for mark–recapture and occupancy analyses (Lonsinger, Gese, Dempsey, & Kluever, 2015; Woodruff, Johnson, & Waits, 2015).

When planning a MIS or noninvasive sampling strategy, it is important to account for patterns of social structure (random or nonrandom association of individuals), habitat‐use and availability of the material produced (e.g., faeces, urine, partially consumed food). This is, in part, because it is important to maximise sampling opportunities for elusive species, given the labour‐intensive nature of field work, but also because certain parameters (e.g., genotype capture‐recapture methods to estimate census size) require the application of assumptions about sampling that may or may not be satisfied if sampling is conducted incorrectly.

Consideration also needs to be given to the most suitable collection and storage method for the study species and sample type. For example, the time since deposition, environmental conditions, part of faeces sampled and storage medium can influence the quality of genotypes obtained, showing the importance of sampling protocol (Stenglein, De Barba, Ausband, & Waits, 2015; Wultsch, Waits, Hallerman, & Kelly, 2016).

### 2.2 Molecular methodologies

The human and agricultural genetics communities have already embraced SNPs for genotyping because of their myriad advantages over microsatellites (although microsatellites are still preferred by some in the human forensics field; Butler, 2015; FAO, 2015). There are many methods for genotyping thousands of SNPs, including variations on RAD‐seq (Andrews, Good, Miller, Luikart, & Hohenlohe, 2016; Baird et al., 2008) and genotyping by sequencing (Elshire et al., 2011). These approaches could be useful in MIS if sufficient DNA can be obtained (e.g., Chiou & Bergey, 2015), but these anonymous‐marker approaches often require considerably more DNA than is typically available to biologists using MIS. The DNA extracted from such samples may also contain xenobiotic environmental DNA (eDNA), often from nontarget organisms, and thus require rigorous postsequencing filtering. Furthermore, these approaches genotype far more loci than needed for individual identification and assessments of relatedness, population structure and other parameters of general interest in genetic monitoring studies and are thus economically inefficient. However, some next‐generation sequencing and advanced genotyping methods are particularly suitable for the low‐quality or quantity of DNA that are typically obtained from MIS; we broadly categorise these into SNP arrays and target enrichment methods. We highlight these methodologies in the subsequent sections, but acknowledge that significant prior sequencing and bioinformatic analyses will be required to identify loci suitable for genotyping MIS samples using these platforms (e.g., Andrews et al., 2016; De Wit et al., 2012; Elshire et al., 2011; Morin et al., 2018). We provide a brief description of the methods and their application to MIS samples, including information on error rates and approximate costings (Table 2).

#### 2.2.1 SNP arrays

Platforms that more efficiently assess relevant numbers of SNPs include the Fluidigm SNPtype assay (Tables 1 and 2). Briefly, the Fluidigm assay uses a two‐stage amplification process with the first pair of primers amplifying the locus containing the SNP and the second pair amplifying specific alleles, integrating distinct fluorescent labels. The Fluidigm platform simultaneously genotypes up to 96 SNP loci in 96 samples, determining the SNP genotype at an individual locus by measuring the fluorescence intensity of both alleles. The fisheries community has embraced SNP genotyping assaying scores of loci with the Fluidigm platform (Bonanomi, Therkildsen, Retzel, & Berg, 2016; Campbell & Narum, 2011; Hauser, Baird, Hilborn, Seeb, & Seeb, 2011), and recently, several wildlife studies have also used this platform in a monitoring context (Table 1: Doyle et al., 2016; Kraus et al., 2015; Nussberger, Wandeler, & Camenisch, 2014). The Fluidigm SNP type assay seems to have relatively low error rates (e.g., 0.2% in DeWoody et al., 2017; 0.4% in Doyle et al., 2016; where error rates are estimated from the number of mismatches between replicates and consensus genotype; 1%–3% per allele in Kraus et al., 2015; 1.7% per locus in Nussberger et al., 2014). The low error rate is important for all aspects of molecular ecology, but particularly for inferences of individual identification, parentage and relatedness. In addition, the Fluidigm platform had a higher genotyping success rate than microsatellite genotyping in hair samples from wolf faeces (87% and 70%, respectively) and wild cat hair (80% vs. 54%) but similar success rates in brown bear hair samples (97% and 99%, respectively; after quality control; von Thaden et al., 2017).

**Table 1:** Contemporary approaches for genotyping low‐quality and/or quantity DNA samples

Reference	Platform/method	Starting material	Species	Inference
SNP Arrays				
Morin and Mccarthy (2007)	Ampliflour SNP genotyping	Bone	Bowhead whale (*Balaena mysticetus*)	Development/validation of SNP markers
Mesnick et al. (2011)	Ampliflour SNP and microsatellite genotyping	Skin	Sperm whale (*Physeter macroephalus*)	Population structure
Nussberger et al. (2014)	Fluidigm	Hair	European wildcat (*Felis silvestris silvestris*)	Validation of SNP markers and studying introgression
Ruegg et al. (2014)	Fluidigm	Feathers	Wilson's warbler (*Cardellina pusilla*)	Tracking migratory populations
Kraus et al. (2015)	Fluidigm	Faeces	Grey wolf (*Canis lupus*)	Development/validation of SNP markers
Norman and Spong (2015)	Fluidigm	Faeces	Brown bear (*Ursus arctos*)	Reconstructing pedigrees and estimating dispersal
Doyle et al. (2016), Katzner et al. (2016)	Fluidigm	Feathers	Golden eagle (*Aquila chrysaetos*)	Population structure, parentage and provenance
Stetz et al. (2016)	Fluidigm	Faeces	River otter (*Lontra canadensis*)	Development/validation of markers, population assignment
Spitzer et al. (2016)	Fluidigm	Faeces	Brown bear (*Ursus arctos*)	Pedigree and population size estimation
DeWoody et al., 2017;	Fluidigm	Skin	Grey whale (*Eschrichtius robustus*)	Individual ID and relatedness
von Thaden et al. (2017)	Fluidigm	Hair and faeces	European wildcat (*Felis silvestris silvestris*); brown bear (*Ursus arctos*); grey wolf (*Canis lupus*)	Validation and population structure analysis
Hoffman et al. (2012)	Illumina GoldenGate genotyping assay	Skin	Antarctic fur seal (*Arctocephalus gazella*)	Development/validation of markers
Monzón et al. (2014)	Illumina GoldenGate genotyping assay BeadXpress platform	Faeces	Coyote (*Canis latrans*)	Admixture and hybridization
Fitak et al. (2015)	MassARRAY (Sequenom)	Faeces	Pumas (*Puma concolor*)	Development/validation of SNP markers
Goossens et al. (2016)	MassARRAY (Sequenom)	Faeces	Asian elephant (*Elephas maximus*)	Population structure and genetic diversity, comparison of SNPs with microsatellites
Fabbri et al. (2012)	SNPs Pyrosequencing (Biotage), SNaPshot (ABI), Taqman (ABI)	Faeces	Grey wolf (*Canis lupus*)	Development/validation of markers
Targeted sequence capture				
Perry et al. (2010)	RNA bait capture/Illumina sequencing (Agilent's SureSelect)	Faeces	Chimpanzees (*Pan troglodytes*)	Validation/SNP genotyping for genetic diversity
Snyder‐Mackler et al. (2016)	RNA bait capture/Illumina sequencing	Faeces	Baboons (*Papio papio*)	Development/validation of markers, pedigree analysis
De Barba et al. (2017)	High‐throughput sequencing of microsatellites (Illumina MiSeq)	Faeces	Brown bear (*Ursus arctos*)	Development/validation of markers
Other examples				
Chiou and Bergey (2015)	ddRAD using FecalSeq	Faeces	Baboons (*Papio papio*)	Development/validation of markers
Russello et al. (2015)	nextRAD	Hair	American pika (*Ochotona princeps*)	Population structure and outlier loci analysis

**Table 2:** Selective summary of characteristics of next‐generation sequencing platforms that could be suitable for low‐quality or quantity DNA templates frequently obtained during MIS projects. Costings are provided in euros (€)

Platform	Development cost	Run cost	Effort (after DNA extraction)	Information	Error rate	DNA required	References
Fluidigm	€4300 for oligos to query 96 SNPs, access to Fluidigm system	€1250 for genotyping 96 individuals at 96 SNPs	PCR	SNP genotype	~1% ^A^	Nanograms	Doyle et al. (2016), Katzner et al. (2016), DeWoody et al. (2017)
Amplifluor	€2200 for oligos for 96 loci, access to qPCR machine	€250 for genotyping 96 samples at 96 loci, based on 20 loci multiplex^a^	PCR and analysis of qPCR results	SNP genotype	1.4% ^B^	Nanograms	Mesnick et al. (2011)
MassARRAY	€2600 for oligos for 96 loci, assuming two alleles per locus, access to MassARRAY system	€777 (384 well format, €8.09 per sample) to €1,376 (96 well format, €14.33 per sample) to genotype 96 individuals at 96 SNPs (24‐loci multiplex)	Multiplex PCR step, clean‐up step, primer extension step and another clean‐up step, run on compact mass spectrometer	SNP genotypes	Faecal sample error rate: 24‐loci multiplex 9%; 42‐loci multiplex error rate: 25% ^A^	Nanograms (10 ng per multiplex reaction recommended)	Goossens et al. (2016)
GT‐seq	<€9000: primary cost is oligos but a pilot study of the markers is suggested, high‐throughput sequencing run	€3.43 per sample, based on example where 2068 samples were genotyped at 192 loci	For each of the 22 × 96‐well plates there were two PCR steps and one normalization step	SNPs; could be extended to haplotypes	0.01%^C^	Nanograms (10 ng for first PCR minimum recommended concentration)	Campbell et al. (2015), N. Campbell, pers. comm.
Microsatellite sequencing	Primary costs are optimisation and validation study, as well as oligonucleotides	€2470 to sequence 96 samples at 14 loci replicated eight times, or €3.20 per replicated PCR product	Multiplex PCR, purification and quantification of pooled PCR product and sequencing run	Microsatellite genotypes	Good quality reference hair: allelic dropout (ADO): 3.9%, false allele rate (FA): 0.3%, Noninvasively collected low‐quality hair: ADO: 10.6%, FA 0.8%; Low‐quality faecal samples: ADO: 13.7%, FA: 0.8%^C^	Not quantified in study, but estimated to be in range of nanograms	De Barba et al. (2017), M. De Barba, pers. comm.

Error rate reported is based on replicate genotyping^A^ or calculated per allele^B^ or per locus^C^.

a Based on purchase of 5,000 assay kit.

A technologically similar, fluorescence‐based platform, Amplifluor SNP genotyping system, has been shown to be highly sensitive with low‐quality/quantity samples: there was a high level of genotyping success with as few as 10 DNA templates per assay (Morin & Mccarthy, 2007). Mesnick et al. (2011) used eight microsatellite loci and 38 Amplifluor SNP loci to investigate the population structure of North Pacific sperm whales (*Physter macrocephalus*). The Amplifluor SNP loci had a comparable error rate (1.4% per allele) to the microsatellite loci (0.9% per allele) in this study (Tables 1 and 2).

In contrast to the fluorescence‐based platforms, the MassARRAY platform uses mass spectrometry to determine SNP alleles. The key difference is the use of the primer extension or iPLEX reaction, which incorporates one mass‐modified nucleotide, depending on the allele and assay design, enabling the detection of single base or small insertion/deletion polymorphisms. A compact mass spectrometer (Sequenom) is then used to infer genotypes based on the position of the peaks in the spectra, corresponding to different alleles at different loci (Gabriel, Ziaugra, & Tabbaa, 2009). The platform has potential for MIS samples: in a recent study, the MassARRAY system successfully genotyped a higher proportion of puma scat samples (59.8%) than a conventional microsatellite genotyping approach (39.9%), with no significant difference in error rates between the methods (Fitak, Naidu, Thompson, & Culver, 2015). However, another study that used both microsatellite genotyping and MassARRAY assays to genotype Bornean elephant blood and faeces found a lower rate of genotyping success and higher error rates for the SNP platform in faecal samples (Goossens et al., 2016). The authors found a trade‐off between genotyping success and multiplexing level, with smaller multiplexes having greater success (Table 1, Goossens et al., 2016), and suggested that the issue could be related to the lower quality of faecal DNA.

#### 2.2.2 Target enrichment methods

The aim of target enrichment is to selectively capture genomic regions of interest before high‐throughput sequencing. Target enrichment methods can be a highly sensitive way of selectively and reproducibly obtaining genomic data. Genomic regions can be selectively targeted using PCR, as well as in‐solution or array‐based methods. PCR‐based methods are suitable for MIS as they typically require only small amounts of starting material and, by utilizing multiplex PCR and combinatorial barcoding techniques, can be cost‐effective. One such method is GT‐seq (Campbell, Harmon, & Narum, 2015; Table 2), which has been used to genotype steelhead trout (*Oncorhynchus mykiss*) to assess abundance, migration timing and stock composition (Hess et al., 2016; Matala et al., 2016). GT‐seq is essentially a massively multiplexed two‐step PCR reaction; in the first PCR reaction, SNP loci are amplified in a multiplex PCR, and in the second reaction, sequencing adaptors and unique identifiers (barcodes) are added to each sample. After Illumina sequencing, the barcodes are used to separate reads into samples, using a custom bioinformatics pipeline (Campbell et al., 2015). The GT‐seq method appears to have a low error rate; the method had a 99.9% concordance rate with genotypes generated with the Fluidigm platform. The method may require additional optimization for low‐quality/quantity DNA samples, although it works well with sheared DNA templates, success rates drop off when DNA concentrations <10 ng/μl (N. Campbell, pers. comm.).

Another targeted PCR approach has focused on the use of high‐throughput sequencing to generate microsatellite genotypes (e.g., De Barba et al., 2017). This approach typically involves PCR amplification of microsatellite loci, followed by multiplexing and high‐throughput sequencing (e.g., De Barba et al., 2017; Vartia et al., 2016). The potential advantages over conventional microsatellite genotyping includes identification of length homoplasy (which can be high, e.g., identified in 38 of 53 loci; Vartia et al., 2016) and cost‐effectiveness at higher numbers of samples and/or markers (Darby, Erickson, Hervey, & Ellis‐Felege, 2016). High‐throughput sequencing of microsatellite genotypes also has the benefit of rapidly generating consensus genotypes using bioinformatic analysis pipelines, either from whole‐genome (e.g., Kistler et al., 2017) or amplicon data (e.g., Suez et al., 2016; Zhan et al., 2017). Furthermore, this approach could have the advantage of linking into legacy data sets if the same sets of loci can be used in the new and traditional microsatellite genotyping platforms. However, optimization and validation steps are required to move microsatellite genotyping on to a new sequencing platform (e.g., De Barba et al., 2017), which can be technically challenging. The application of this microsatellite genotyping with high‐throughput sequencing to MIS studies has been limited thus far. However, De Barba et al. (2017) found that a set of microsatellite loci optimised for high‐throughput sequencing increased the yield and accuracy of genotypes generated from faecal samples, compared with metrics previously reported for genotyping microsatellites from faecal samples with capillary electrophoresis.

DNA capture methods, in conjunction with high‐throughput sequencing, have been used to investigate phylogenetic questions (e.g., Hancock‐Hanser et al., 2013), but the application of such methods to within‐population studies has been limited thus far. One successful example was the use of custom biotin‐tagged RNA baits to capture genomic DNA from faecal samples from 62 wild baboons (*Papio papio*). The enriched libraries were sequenced with Illumina HiSeq and provided sufficient genomic markers to undertake pedigree reconstruction (Snyder‐Mackler et al., 2016). Another study, using bait captures generated from the Agilent SureSelect system, successfully sequenced more than 1.5 Mb of nuclear DNA and the entire mitochondrial genome from chimpanzee faeces (Perry, Marioni, Melsted, & Gilad, 2010). These studies highlight the potential of bait capture approaches, both custom and using a commercial provider, in a genetic monitoring context. Such approaches could be aided by the use of novel methods that enrich samples for endogenous DNA, such as FecalSeq (Chiou & Bergey, 2015).

### 2.3 Data analysis

SNP array platforms have proprietary software packages that are used to score genotypes and often provide a degree of confidence in genotype calls (e.g., Sequenom platform). Such automated calling is not always accurate, and it is recommended that researchers visually check the data for error. This is particularly true for noninvasively collected samples, which can have higher error rates (e.g., Bayerl et al., 2017). Target capture approaches that use high‐throughput sequencing tend to have custom bioinformatics pipelines (e.g., Campbell et al., 2015). However, the major steps are similar between studies and include filtering of reads based on quality scores and demultiplexing reads into samples and loci. Genotyping is then conducted using custom bioinformatics tools and information such as the relative frequency and read depths of sequences likely to be alleles versus PCR/sequencing artefacts (Campbell et al., 2015; De Barba et al., 2017).

### 2.4 Quality control

Genotype data are imperfect and subject to missing genotypes (errors of omission) as well as erroneous genotypes (errors of commission; Faria et al., 2011). Missing and erroneous genotypes can be due to many possible causes, such as suboptimal genotyping protocols, limited DNA quantity and quality, contamination and human error (Bonin et al., 2004; Pompanon, Bonin, Bellemain, & Taberlet, 2005). MIS data are especially problematic due to the low DNA quality and quantity, and can incur a high rate of error.

Missing and erroneous genotypes affect many genetic analyses, yielding potentially biased and imprecise results and, in turn, incorrect conclusions. Broadly speaking, analyses that use genotype data are more severely impacted than analyses that use allele frequency data. For example, genetic differentiation, measured by F_ST_ (Wright, 1931) and evaluated by several estimators (Nei, 1973; Weir & Cockerham, 1984), is determined by marker allele frequencies. As missing and erroneous genotypes do not substantively change allele frequencies, such errors tend to have small effects on F_ST_. In contrast, genotype‐based analyses, such as inferences of identity, relatedness and relationship, are strongly influenced by data quality. Ignoring or underestimating genotyping errors can lead to false parentage exclusions (Dakin & Avise, 2004; Wang, 2010), false sibship exclusions (Wang, 2004), false exclusion of duplicated individuals and thus overestimation of population size (Creel et al., 2003; Waits & Leberg, 2000).

The impact of missing and erroneous genotypes also depends on how they are distributed among loci and among individuals. The best scenario is a uniform distribution, such that no specific loci and no specific individuals are too problematic to be useful. However, with MIS samples, missing and erroneous genotypes are usually clustered among individuals because the sample DNA quality and quantity can differ substantially among samples, and error rates have been shown to vary considerably across loci (Broquet & Petit, 2004; Campbell et al., 2015; Gagneux, Boesch, & Woodruff, 1997; Paetkau, 2003).

A source of error common to both microsatellites and SNP genotypes is allelic dropout (Bayerl et al., 2017; Gagneux et al., 1997). This is where a heterozygous genotype may be incorrectly typed as a homozygote. Allelic dropout is generally caused by random effects that result in missing one of the two alleles at a diploid locus. It is strongly correlated with lower coverage (5–20×; Nielsen, Paul, Alberechtsen, & Song, 2011) for SNPs from next generation sequencing (NGS). Loci can also have null alleles, which produce no observable phenotype (Dakin & Avise, 2004). Thus, null allele homozygotes would be scored as missing data, whereas a null allele heterozygote would be scored (erroneously) as a homozygote of the observable allele. Traditionally, a single best genotype is reported for an individual at a locus. The large uncertainties of such called SNP genotypes mean that erroneous results could be produced, such as an overestimation of inbreeding (Vieira, Fumagalli, Albrechtsen, & Nielsen, 2013) and biased estimates of relatedness (Vieira, Albrechtsen, & Nielsen, 2016), just as it can in standard genetic markers such as microsatellites (Bonin et al., 2004).

The best practice now is to call all possible genotypes at a SNP locus with corresponding likelihoods that summarise the quality and evidence of the reads data, as well as incorporating information on population‐level allele frequencies (Nielsen et al., 2011). Using genotype likelihoods to account for uncertainties at the individual genotype level, an appropriately designed programme can yield unbiased and accurate estimates of parameters such as inbreeding and relatedness (Vieira et al., 2013, 2016), even when the average coverage is very low, and thus, the genotype data are highly uncertain (Buerkle & Gompert, 2013). Buerkle and Gompert (2013) show that partitioning the sequencing effort maximally among individuals and obtaining approximately one read per locus and individual (1× coverage) yields the most information about a population. More statistical methods urgently need to be adapted or developed to take advantage of genotype likelihoods. One obstacle is computational burden, which increases enormously by considering three possible rather than a single genotype at each locus for each individual, although increasingly sophisticated algorithms and parallelization may mitigate this issue.

The fundamental strategy for improving data quality is by enhancing DNA quantity and quality, reducing contamination, improving PCR protocols (or NGS coverage), employing good laboratory practices and other technical improvements that are beyond the scope of this review (for more information: Bonin et al., 2004; Morin et al., 2010; Paetkau, 2003; Pompanon et al., 2005; Waits & Paetkau, 2005). As with microsatellite genotyping (Bonin et al., 2004), the best practice is to report error rates or genotype likelihoods from SNP genotype studies. There are two categories of mistyping rate estimation. One category is based on duplicated genotype data (i.e., an individual is genotyped independently multiple times at a locus), measuring the consistency of repeated genotypes (e.g., Broquet & Petit, 2004) or estimating the error rates of repeated genotypes (e.g., Johnson & Haydon, 2007; Zhan, Zheng, Bruford, Wei, & Tao, 2010). These methods generally overestimate the mistyping rate of the final genotype data set, because repeated genotyping allows for the detection and elimination of such errors in the final consensus genotypes. This has been a common method for reporting genotype error rates in many SNP array studies (Table 2).

The second category for estimating mistyping rates is based on the final consensus genotypes and is accomplished by examining the genotype against either the Hardy–Weinberg equilibrium (e.g., Hosking et al., 2004) or the Mendelian segregation law in a known (e.g., Sobel, Papp, & Lange, 2002) or reconstructed pedigree (e.g., Wang & Santure, 2009). The former is effective only in detecting null alleles and allelic dropouts that can cause directional deviations from Hardy–Weinberg proportions (i.e., an excess of homozygotes), but is ineffective for mistypings that do not cause detectable distortions, such as false alleles. This error estimation approach can have low power (e.g., Cox, 2006), and relies on the absence of confounding factors, such as strong selection, inbreeding and population structure. Some methods have been developed to make joint estimates of null allele frequencies and inbreeding (e.g., Hall, Mercer, Phillips, Shaw, & Anderson, 2012). How well such methods work has not been thoroughly evaluated, however.

Pedigree, either known or inferred, can be used in likelihood methods to detect erroneous genotypes and to estimate mistyping rate at each locus (Sobel et al., 2002; Wang, 2009). These methods can be used to infer null allele rates, allelic dropout rates and false allele rates and are highly robust to the violations of some common assumptions such as random mating and the absence of inbreeding population structure. Such mistyping estimation methods, together with data missing rates, measure data quality. More importantly, these methods allow downstream analyses to effectively filter out the noises in extracting information from the genotype data and in arriving at robust and accurate analysis results (e.g., Kalinowski, Taper, & Marshall, 2007; Wang, 2004).

## 3 QUESTIONS AND METRICS THAT CAN BE INVESTIGATED WITH MIS

The power of genetic monitoring using MIS is the range of questions that can be addressed. Here, we discuss how environmental samples can be used to address broad questions, such as species occupancy range, and how individual‐level MIS samples (e.g., feathers, faeces) can be used to estimate individual‐ and population‐level parameters, such as vital rates and population genetic parameters.

### 3.1 Environmental samples

#### 3.1.1 Occupancy and range

Species and site occupancy and presence/absence analysis relies on information needed to avoid biased estimates; quantifying detection rates and especially understanding whether a target species is present, but undetected (e.g., MacKenzie, Nichols, Hines, Knutson, & Franklin, 2003). Molecular data can augment these studies, enabling more accurate detection even at very low levels of occupancy using environmental samples and DNA barcoding (e.g., Boothroyd, Mandrak, Fox, & Wilson, 2016) or faecal samples of uncertain species identity (e.g., Faria et al., 2011; Palomares et al., 2002; Stanton et al., 2016), although its use is again severely constrained by DNA quality considerations. For example, Stanton et al. (2016) assayed faecal samples from an unsurveyed region in the Democratic Republic of Congo for the presence of okapi (*Okapia johnstoni*). Of the 24 faecal samples detected, only 12 yielded DNA but of these six were identified as okapi and these yielded four mitochondrial haplotypes (hence allowing the inference of minimally four individuals being present). Advances in environmental DNA (eDNA) analysis are enhancing our ability to examine past and present distribution and diversity of various species and communities (see Box 2).

Box 2 **Environmental DNA (eDNA) in the genetic monitoring context**

1 Genomic sequencing technologies are broadening the scope of eDNA studies in genetic monitoring. Researchers have demonstrated that ecological research questions can be addressed using DNA extracted from water, soil, sediments, snow, browsed foliage, as well as invertebrates (“iDNA”; Schnell et al., 2015) that feed on species of interest: some examples are illustrated below.

**Figure 2:**
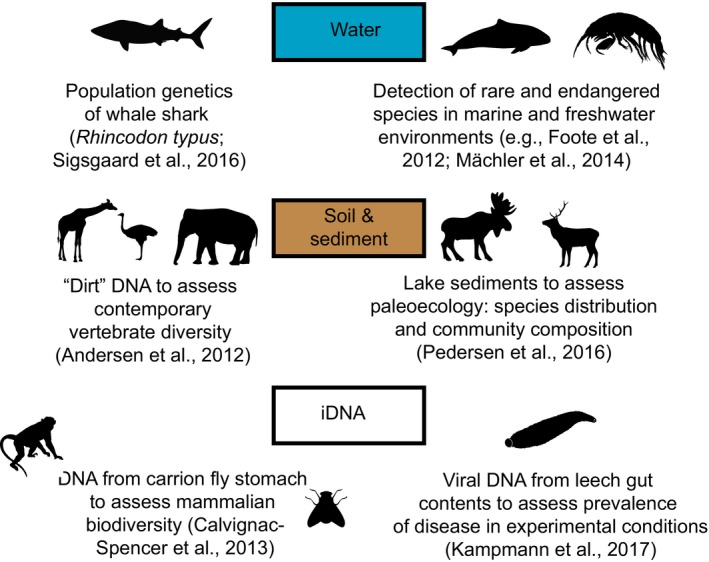

### 3.2 Individual‐level samples

#### 3.2.1 Individual identification and its application: Abundance/density

The recapture of individuals, identified by their genotype, across time and space, has allowed genetic monitoring to become a key tool in estimating abundance, density and demographic parameters in a variety of species. It has been particularly important in species that are evasive, endangered (Taberlet et al., 1997), dangerous (Kendall et al., 2009) or otherwise difficult to capture/recapture (Constantine et al., 2012), such as those that show limited variation in natural markings, reducing the usefulness of conventional identification from photographs (e.g. juvenile cetaceans, Carroll et al., 2016). The use of genetic monitoring to estimate abundance ranges from the enumeration of the number of genotypes in a region (Taberlet et al., 1997), to single‐session models (Miller, Joyce, & Waits, 2005; Petit & Valière, 2006), to occupancy (Lonsinger, Gese, Bailey, & Waits, 2017; Marucco, Avanzinelli, & Boitani, 2012), to complex mark–recapture models that integrate sex, age and reproductive status information (Carroll et al., 2013; Woodruff et al., 2016). The advent of spatial mark–recapture models (Efford, 2004, 2011; Royle & Young, 2008) has improved analytical tools for density estimates using genetic monitoring approaches (Mollet, Kéry, Gardner, Pasinelli, & Royle, 2015; Russell et al., 2012; Thompson, Royle, & Garner, 2012).

Historically, population estimation in genetic monitoring has relied on individual identification using microsatellite loci. Recognition that genotyping error, correlated with low‐quality DNA templates, can create large biases in population abundance estimates (Waits & Leberg, 2000) has required the development of methods that generate consensus genotypes from multiple PCR replicates (Taberlet et al., 1997) or models that directly incorporate genotyping error (Lukacs & Burnham, 2005; Wang, 2016). In transitioning to the genomics era, new approaches such as direct sequencing of microsatellite loci (De Barba et al., 2017) and SNP analysis will be used (Fitak et al., 2015; Kraus et al., 2015). Large panels of markers from next‐generation sequencing will allow for the more efficient identification of related individuals. This will allow the use of close kin mark–recapture models, which extend the idea of using the recapture of individuals to the recapture of close kin to estimate demographic parameters such as effective population size (Bravington, Skaug, & Anderson, 2016; Wang, 2009).

#### 3.2.2 Other demographic parameters

Long‐term effective management of populations and species requires sound knowledge of key demographic parameters, such as survival and growth rates. The most common way to estimate such parameters is from long‐term studies that follow individuals over time (McClintock, White, Antolin, & Tripp, 2009). Long‐term MIS studies have been an effective way to estimate survival and growth rates in a range of species, by tracking individuals using their genotypes. This has been accomplished using mark–recapture models in species such as southern right whales (*Eubalaena australis*; Carroll et al., 2013, 2016), the dendrobatid frogs (*Allobates femoralis*; Ringler, Mangione, & Ringler, 2015), Māui dolphins (*Cephalorhynchus hectori maui*; Baker et al., 2013), brown bears (Tenan et al., 2016) and imperial eagles (*Aquila heliaca*; Rudnick et al., 2005). The definitive DNA marks provided by genetic monitoring can provide robust population estimates in age‐structured populations that can be difficult to observe in the wild. The difference between observational and MIS genetic population estimates can have profound impacts on demographic models and associated conservation actions (Katzner, Ivy, Bragin, Milner‐Gulland, & DeWoody, 2011).

#### 3.2.3 Individual space use and movement

Genetic monitoring using MIS can also provide valuable information on individual space use, movement patterns and dispersal. This approach has been used to monitor population expansion and individual dispersal distances in reintroduction efforts for brown bears (De Barba, Waits, Garton, et al., 2010), grey wolves (*Canis lupus*; Stenglein et al., 2010) and Columbia Basin pygmy rabbits (*Brachylagus idahoensis*; Demay, Becker, Rachlow, & Waits, 2017), investigate connectivity between migratory habitats in humpback whales (*Megaptera novaeangliae*; Constantine et al., 2014; Garrigue et al., 2011), to monitor roosting movements in eagles (Rudnick et al., 2008) and to detect natural range expansion (Carroll et al., 2014; Valière et al., 2003) using microsatellites. MIS using microsatellites has also been valuable for assessing the effectiveness of corridors (Dixon et al., 2006) and evaluating potential barriers (Epps et al., 2005; Kendall et al., 2009; Proctor et al., 2005). More recently, SNPs have been utilised to estimate pedigree‐based dispersal models in brown bears (Norman & Sprong, 2015) and to infer individual provenance (i.e., identify potential migrants) based on the distribution of pairwise relatedness (DeWoody et al., 2017).

#### 3.2.4 Relatedness and kin structure (kinship)

Since the development of relatively large panels of markers (microsatellites and more recently SNPs), those panels have been used to monitor the existing relationships between individuals of a given population, either to investigate genetic and social structure, gene flow, reconstruct pedigrees or minimise inbreeding (Caniglia, Fabbri, Galaverni, Milanesi, & Randi, 2014; Da Silva, Lalonde, Quse, Shoemaker, & Russello, 2010; Jones et al., 2002; Peters, Queller, Imperatriz‐Fonseca, Roubik, & Strassmann, 1999; Stenglein, Waits, Ausband, Zager, & Mack, 2011). Metrics generally used to measure relatedness between two individuals estimate either a summary statistic (such as coancestry coefficient and its equivalents), which would correspond to the relatedness between two individuals, or the probability that two individuals are linked with a particular relationship (parent–offspring, first cousins, self‐outbred sibs, etc.) given the data (Wang, 2011). In some cases, the reliability of relatedness estimates can be limited, especially when the population under study exhibits low genetic variation for the marker set; therefore *a priori* simulations should be performed to select the most appropriate estimator and assess its accuracy (Glaubitz et al., 2003; Taylor, 2015). The development of NGS tools is expected to increase the availability of high‐density panels, thus improving the reliability of estimators. It may also allow the use of new metrics, such as chromosome‐segment‐based ones, considering the measurement of coancestries based on shared segments of identity by descent, instead of averaging, marker by marker, the probability that two alleles are identical in state (De Cara, Villanueva, Toro, & Fernández, 2013).

### 3.3 Population genetic parameters

#### 3.3.1 Genetic diversity

Historically, microsatellites were used with MIS to produce estimates of population genetic variation based on allelic diversity and heterozygosity. Allelic diversity, which is often high and variable among microsatellite loci, is not very informative for SNPs. This is because SNPs have comparatively few alleles, generally limited to one or two (i.e., third or fourth alleles at a locus do not materialise before one of the original two is lost due to drift or selection).

On the other hand, estimates of heterozygosity using SNP loci can be more informative than microsatellites because the additional SNP loci surveyed provide higher precision. For example, Doyle et al. (2016) surveyed 162 SNPs in golden eagles and found that mean observed heterozygosity (H_O_) was 0.32 ± 0.01 in juveniles whereas adult H_O_ was 0.35 ± 0.01, a significant statistical difference consistent with expectations of viability selection. Unfortunately, the types of SNP arrays often used in MIS studies preclude the evaluation of other genetic diversity metrics that will likely be important in the future (e.g., runs‐of‐homozygosity or copy number variants, see Leroy et al., 2017). This is a factor worth considering when planning a study, as evaluating change in genetic diversity metrics over time is an important task of genetic monitoring (see Box 3).

Box 3 **The importance of “delta” in genetic monitoring**

1 Endangered species are, by definition, the subject of local, regional, national and international legislation, including the Convention on Biological Diversity (CBD). The CBD's 2020 Targets include a commitment to “*minimise genetic erosion”* and “*safeguard genetic diversity”* (Bruford, Davies, Dulloo, Faith, & Walters, 2017; Hoban et al., 2013). These commitments require a means of verification and imply a reference point from which to determine changes, or “delta,” in genetic diversity. The statistical approaches needed to evaluate changes in genetic diversity over short timescales, however, require development. Temporal genetic monitoring of species at the same location has been accomplished in a some well‐studied populations or species of high conservation concern (e.g., Italian brown bears; De Barba, Waits, Garton, et al., 2010; Māui dolphins; Baker et al., 2016) or where hybridization is a threat (e.g., red wolves and coyotes; Bohling et al., 2016).

In the absence of samples from a population over time, analysis of genetic data using single point samples can provide insights into recent demographic change (e.g., Goossens et al., 2006). However, single point estimators can have wide variance and provide inconsistent values depending on the methods chosen or model assumptions (Barker, 2011). To aid understanding of which metrics would be the most sensitive to detecting short‐term declines in genetic diversity, Hoban et al. (2014) carried out an assessment of temporal indicators of genetic erosion (*sensu* Aichi Target 13).

The number of alleles per genetic locus (K) outperformed all other potential indicators across all scenarios. However, the power with which to detect a decline in diversity in K varied with more samples or markers with, for example, 2500 SNPs being effective at detecting minor demographic declines after 8–10 generations (see Figure). Hoban et al. (2014) also found that statistical power to detect change improved if samples were available before the onset of decline, implying that archived and museum collections can clearly play an important role as part of monitoring programmes.

**Figure 3:**
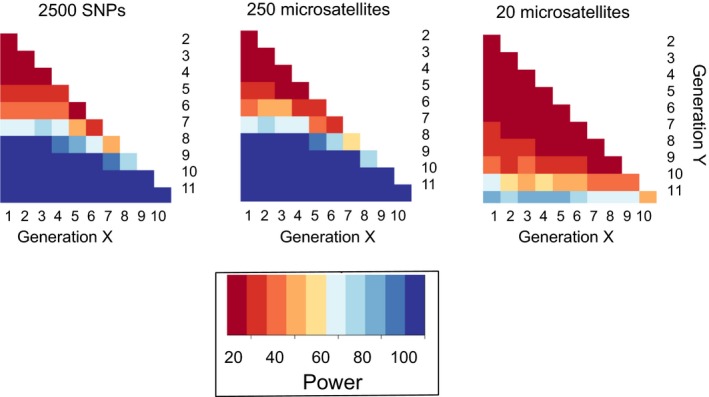

Figure: Modified from Hoban et al. (2014): Comparison of the proportion of 100 replicates (i.e., power) in which the indicator, K (alleles per locus), was significantly different between generations X and Y. The scenario simulates a population that has experienced an exponential decline of 97%, using different types and numbers of genetic markers. The darkest blue is power >0.90; power <0.50 is orange; and power <0.10 is dark red.

#### 3.3.2 Effective population size

Populations of conservation concern are usually small and thus experience inbreeding and genetic drift that could lead to a depletion of genetic variation. The parameter effective population size measures the strength of the stochastic processes of inbreeding and genetic drift (Wright, 1931) in a population. It is defined as the size of an idealised population which would give rise to the same rate of inbreeding or drift as observed in the actual population under consideration (Caballero, 1994; Wang, Santiago, & Caballero, 2016). For wild populations where pedigree data are unavailable, marker data, generated from MIS, can be used to estimate both historical (e.g., Beerli & Felsenstein, 2001) and recent/current effective size of a population (Wang, 2016). Recent effective population size can be estimated by approaching a wide range of signals (temporal variance in allele frequency, frequency of close relatives, linkage disequilibrium, heterozygosity excess, etc.) measuring either inbreeding or genetic drift in a given time period. By consequence, depending on the data available, various approaches can be implemented, each with its own advantages and limits. For instance, linkage disequilibrium estimates of contemporary effective population size can be obtained from unlinked microsatellites or SNPs. When the linkage information between SNPs is also available, linkage disequilibrium estimates allow the inference of effective population size over past generations (Hayes, Visscher, Mcpartlan, & Goddard, 2003).

While this approach has its limitations and caveats (Palsbøll, Peery, Olsen, Beissinger, & Bérubé, 2013), MIS has been used to estimate long‐term effective population sizes in species such as southern right whales (Carroll et al., 2015) and Sumatran orangutans (*Pongo abelii*; Nater et al., 2013). Historical samples can provide a direct way of assessing past levels of genetic diversity and effective population size and therefore any recent changes in these metrics. Although not typically undertaken using MIS, such studies provide important management information for species of conservation concern, for example, museum specimens were used to assess historical diversity in species such as brown bears (*Ursos actos*; Miller & Waits, 2003) and Seychelles warbler (*Acrocephalus sechellensis*; Spurgin et al., 2014).

Contemporary estimates of effective population size or number of effective breeders are also a critical indication of the genetic resilience of a population (Frankham, Bradshaw, & Brook, 2014), and have been estimated with MIS for brown bears (De Barba, Waits, Garton, et al., 2010; Gonzalez et al., 2016), Hector's dolphin (*Cephalorhynchus hectori*; Hamner, Constantine, Mattlin, Waples, & Baker, 2017), Māui dolphin (Baker et al., 2016) and Eurasian otters (*Lutra lutra*; Koelewijn et al., 2010). For the purpose of genetic management of endangered species, the current or contemporary effective size is more relevant than historical or long‐term effective size (Wang, 2016).

#### 3.3.3 Social and genetic structure

In addition to the presence/absence and censusing of individuals, additional information can be gained from MIS studies on the socio‐genetic structure of the population being surveyed. This has become a necessity in certain fields (especially in primatology) where, even if individuals can be observed and identified, invasive sampling is regarded as unethical and is often prohibited. Such studies may allow identification of social group‐mediated genetic structure and inferences on sex‐biased dispersal and how these may be modified by habitat fragmentation (e.g., Minhos et al., 2016) and/or hunting and exploitation (e.g., Ferreira da Silva et al., 2014). Understanding social structure and spatial assortment of related individuals using MIS is also an important factor underpinning the accuracy of capture–recapture molecular censusing (Miller et al., 2005; Zhan et al., 2006).

Both in socially structured and unstructured species, population boundaries may spatially coincide with a sampling area being studied using MIS methods. In such cases, it is important to know where these boundaries lie in order to infer the underlying demographic processes structuring the population(s), and to assign individuals to those populations using the correct allele frequency data. Over recent years, numerous studies have successfully investigated genetic structure in wild populations using MIS (e.g., Norman et al., 2017; Russello, Waterhouse, Etter, & Johnson, 2015; Steyer et al., 2016). Different approaches have been developed to investigate the genetic structuring of a group or population, using either multivariate analysis (Jombart, Pontier, & Dufour, 2009) or Bayesian methods for optimizing population features such as Hardy–Weinberg equilibrium (Pritchard, Stephens, & Donnelly, 2000) and even allowing for the integration of environmental and spatial data for interpretation purposes (e.g., Caye, Deist, Martins, Michel, & Francois, 2016; Guillot, Mortier, & Estoup, 2005). Further, these structure‐based approaches are relatively robust in the face of bias related to small sample size or even genotyping error (Smith & Wang, 2014).

#### 3.3.4 Hybridization and introgression

For some species, hybridization and introgression are major threats to population and species persistence creating a need for long‐term genetic monitoring (Allendorf, Leary, Spruell, & Wenburg, 2001). Genetic monitoring approaches using MIS have been applied to detect hybridization in multiple carnivore species including grey wolves (Caniglia et al., 2014; Godinho et al., 2015; Kopaliani, Shakarashvili, Gurielidze, Qurkhuli, & Tarkhnishvili, 2014; Monzón, Kays, & Dykhuizen, 2014), Eastern wolves (*Canis lycaon*, Benson, Patterson, & Wheeldon, 2012), red wolves (*Canis rufus*; Adams et al., 2003; Bohling et al., 2016) and wildcats (*Felix silvestris silvestris*; Anile, Ragni, Randi, Mattucci, & Rovero, 2014; Steyer et al., 2016). The majority of these studies have used mitochondrial DNA and microsatellite markers, but a few have used SNPs to detect hybridization or monitor grey wolves (Kraus et al., 2015; Monzón et al., 2014) and hybridization between wildcats and domestic cats (Nussberger et al., 2014; Oliveira et al., 2015).

### 3.4 Nontarget DNA: Diet

DNA metabarcoding combined with high‐throughput sequencing has proven to be an effective genetic monitoring tool to characterise diet (Pompanon et al., 2012; Valentini et al., 2009). This method has been used to noninvasively study diet in a diverse range of species including Adelie penguins (*Pygoscelis adeliae*; Jarman et al., 2013), golden‐crowned sifaka (*Propithecus tattersalli*; Quéméré et al., 2013), subterranean rodents (*Ctenomys* sp.; Lopes et al., 2015), tapir (*Tapirus terrestris*; Hibert et al., 2013), brown bears (De Barba et al., 2014; Elfström et al., 2014; Valentini et al., 2009), golden marmots (*Marmota caudata*; Valentini et al., 2009), African herbivores (Kartzinel et al., 2015), Hawaiin tree snails (*Achatinella* spp.; O'Rorke et al., 2015; Price, O'Rorke, Amend, & Hadfield, 2017), red deer (*Cervus elaphus*; Fløjgaard, De Barba, Taberlet, & Ejrnæs, 2017) and leopard cats (*Prionailurus bengalensis*; Shehzad et al., 2012). While technical limitations mean that diet inference is typically semi‐quantitative (De Barba et al., 2014; Deagle, Chiaradia, Mcinnes, & Jarman, 2010; Pompanon et al., 2012), the ability to identify primary dietary components is useful for comparative ecological studies (although see Thomas, Deagle, Eveson, Harsch, & Trites, 2016; Thomas, Jarman, Haman, Trites, & Deagle, 2013 for advances in quatitative methods). Furthermore, metagenomic approaches whereby shotgun sequencing is used to characterise both prey and potential pathogens in faecal samples holds the potential to simultaneously characterise diet and microbiomes, while avoiding some of the earlier technical limitations (Srivathsan, Ang, Vogler, & Meier, 2016). In a broader context, assaying dietary niche through genetic monitoring techniques is likely to play a future role in determining the vulnerability of populations to disturbances (Clare, 2014) and is already aiding the restoration and relocation plans for endangered species (Price et al., 2017).

## 4 PAST AND FUTURE OPPORTUNITIES

### 4.1 Legacy data sets

The sheer abundance of microsatellite data sets associated with MIS conservation studies is impressive. Thus, it would be desirable if future monitoring efforts could tie an individual's established microsatellite DNA profile to a new SNP profile. Many individuals of long‐lived species like trees, whales, bears or eagles have already been genetically tagged using microsatellites. In an ideal world, a new DNA profile generated with SNPs would be matched to those generated previously with microsatellites. Unfortunately, this is time‐consuming and expensive because it would require SNP genotyping a reference sample for each individual or having a way to link the SNP genotype to the microsatellite genotype. In principle, it might be possible using a high‐density SNP array to genotype individuals at each microsatellite locus. However, in practice, this depends on the availability of the SNPs, the extent of linkage disequilibrium, recombination rates, nucleotide substitution rates, effective population size, as well as the practicalities of designing assays for the repetitive genomic regions that microsatellites represent. In practice, it is an easy decision to forego microsatellites and establish a new SNP array when monitoring a “new” species. There are online tools, such as the ConGress website that contains a Decision Making Tool, that can help managers to use power analyses to identify optimal methods for a MIS analysis ( http://www.congressgenetics.eu).

In those cases with extensive legacy data sets, it might make the most sense to use “microsatellite sequencing” techniques (e.g., De Barba et al., 2017) in an effort to continue surveying the same loci (albeit with a different technology), at the same time as expanding genome sampling. It may be possible to impute genotypes if sufficiently large sample sizes are available for present and past data, and both legacy and modern platforms, as is routinely carried out for individual types using different SNP panels in livestock species (e.g., Druet, Schrooten, & de Roos, 2010). As an example from cattle, the imputation of 12 microsatellite markers was conducted using a set of 982 SNPs, located within 500 kb of the targeted microsatellite markers (McClure, Mullen, & Kearney, 2014; McClure, Sonstegard, Wiggans, & Van Tassell, 2012).

Such imputation is likely to be far more difficult in wild species that lack pedigrees and dense marker panels. That said, it might be possible to use known or suspected relationships among individuals (e.g., full‐siblings) to leverage microsatellite‐based fingerprinting against SNP‐based fingerprinting.

### 4.2 Future directions

Evolving technology means that genetic monitoring of populations is expanding beyond genotypes. We broadly categorise these methods into those that will help enhance understanding of population demography, health and “functional” or adaptive genetic monitoring. The latter moves beyond using neutral alleles for individual identification and estimation of population genetic parameters to assay loci linked to processes such as inbreeding and adaptation (Table 3). Wildlife forensics is also set to benefit from technological advances (see Box 4).

**Table 3:** Beyond genotypes: selected examples of the application of genomic sequencing technology to study ecology and evolution of species using minimally invasive samples

Reference	Inference	Platform/method	Starting material	Species
Assessing genetic diversity				
Hans et al. (2015)	Diversity of MHC loci	Pooled PCR amplicon sequencing on Illumina MiSeq	Faeces	Gorilla (*Gorilla gorilla*)
Ang et al. (2016)	Diversity of mtDNA	Pooled PCR amplicon sequencing on Illumina HiSeq	Faeces	Tonkin snub‐nosed monkey (*Rhinopithecus avunculus*)
Sigsgaard et al. (2016)	MtDNA haplotype diversity and identity	Illumina MiSeq (bulk sequencing)	eDNA water sample	Whale shark (*Rhincodon typus*)
Health/diet/demography				
Valentini et al. (2009)	Diet	PCR amplicons sequencing 454 platform	Faeces	Golden marmots (*Marmota caudata*) and brown bears (*Ursus arctos*)
Shehzad et al. (2012)	Diet	Pooled PCR amplicon sequencing on Illumina	Faeces	Leopard cat (*Prionailurus bengalensis*)
Jarman et al. (2013)	Diet	Pooled PCR amplicon sequencing on Ion Torrent	Faeces	Adelie penguin (*Pygoscelis adeliae*)
Quéméré et al. (2013)	Diet	Pooled PCR amplicon sequencing on Illumina	Faeces	Golden‐crowned sifaka (*Propithecus tattersalli*)
De Barba et al. (2014)	Diet	Pooled PCR amplicon sequencing on Illumina	Faeces	Brown bear (*Ursus arctos*)
Kartzinel et al. (2015)	Diet and niche partitioning	Pooled PCR amplicon sequencing on Illumina	Faeces	Seven large mammalian herbivores
O'Rorke et al. (2015); Price et al. (2017)	Diet and niche partitioning, environmental restoration planning	Pooled PCR amplicon sequencing on Illumina	Faeces	Hawaiian tree snails (*Achatinella* spp.)
Srivathsan et al. (2016)	Diet and gut parasite characterization	mtDNA shotgun sequencing Illumina HiSeq	Faeces	Banded leaf monkey (*Presbytis femoralis*)
Apprill et al. (2017)	Characterization of respiratory microbiome	Pooled PCR amplicon sequencing on Illumina	Exhaled breath samples	Humpback whale (*Megaptera novaeangliae)*
Raverty et al. (2017)	Genetic monitoring of respiratory microbiome	PCR amplicon sequencing of bacterial DNA barcodes and direct culture of bacteria	Exhaled breath samples	Killer whale (*Orcinus orca*)
Polanowski et al. (2014)	Estimate of chronological age	Bisulphite conversion of PCR products and PYROMARK 24 Pyrosequencing platform sequencing (Qiagen)	Remote skin biopsy sample	Humpback whale (*Megaptera novaeangliae*)

Box 4 **Wildlife Forensics**

1

**Figure 4:**
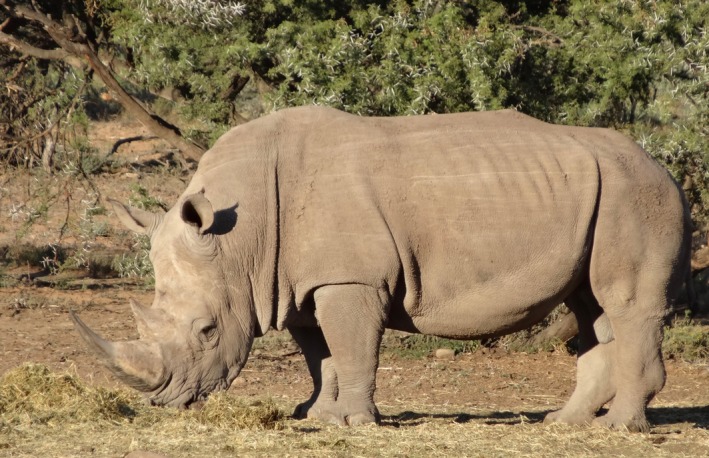

Image: White rhinoceros (*Ceratotherium simum*). Credit: J. A. DeWoody.

As the global threat of illegal wildlife trade becomes more apparent, the use of genetic and genomic tools in the fight against wildlife crime has increased substantially (Corlett, 2017; Ogden & Linacre, 2015; Staats et al., 2016). Traditional genetic tools are increasingly being applied to forensic casework involving material inherently lacking in viable genetic material, for example, microsatellite markers to locate the likely origin of seized elephant ivory (e.g., Wasser et al., 2015), and similar tools are now in routine use to enable the development of a database to allow the matching of carcasses and seized, poached African rhinoceros horn (Harper, Vermeulen, Clarke, De Wet, & Guthrie, 2013). DNA barcoding is being increasingly used in the identification of traded products to species, such as pangolin scales (Mwale et al., 2017), as well as estimating the number of whales traded in meat markets (Baker et al., 2007).

The use of genomics has, however, opened up the possibility of additional applications in the forensic field, including the development of simple, cost‐effective tools to analyse extremely problematic samples and to address questions that were otherwise statistically unattainable using standard genetic approaches. For example, it is possible to identify putrid bushmeat samples, which can be highly degraded once seized, to species level or beyond using low‐cost microarrays (e.g., Rönn et al., 2009). A landmark paper in 2011 developed a set of SNPs for investigation of false eco‐certification of exploited European fish stocks using population assignment that relies on divergent SNPs under the influence of selection in species in otherwise undifferentiated populations, where standard microsatellite‐based population assignment had proved impossible (Nielsen et al., 2012). Furthermore, portable sequencing devices, such as the MinIon (Oxford Nanopore Technologies), are starting to be used to sequence samples in field laboratory conditions (Edwards, Debbonaire, Sattler, Mur, & Hodson, 2016; Quick et al., 2016). This leads to the possibility that in the near future researchers will be able to undertake real‐time assessments of the species, and potentially population of origin, of products in markets.

#### 4.2.1 Population demography

Estimating the chronological age of individuals through genetic monitoring would provide broader insights into population dynamics. Age classes, or the chronological age of individuals in a population, are a critical component to estimating past and future growth rates, as well as population‐level responses to biotic (e.g., prey resources) and abiotic (e.g., hunting) pressures. Conventionally, longitudinal studies that track individuals in well‐studied populations have been the only way to estimate age for many species (Clutton‐Brock & Sheldon, 2010). However, molecular age biomarkers (MAB), those derived from measurable changes in DNA or RNA abundance or sequence change, offer a new way to estimate chronological age. One MAB that held promise was telomeres, and although it has been found to work well in some bird species (e.g., Haussmann, Vleck, & Nisbet, 2003), its wider applicability has been limited (Dunshea et al., 2011). A recent paper showed that epigenetic markers can be used to estimate age in humpback whales (Polanowski, Robbins, Chandler, & Jarman, 2014), using MIS, an approach that has promise in other species (Jarman et al., 2015).

Epigenetic markers might have utility in monitoring other facets of population demography, as epigenetic changes have been linked to early life conditions (Gapp, von Ziegler, Tweedie‐Cullen, & Mansuy, 2014), reproductive maturity (Lomniczi et al., 2013), survival (Fairlie et al., 2016) and response to chemical or physical stressors (Feil & Fraga, 2012), in a variety of species. The development of epigenetic markers therefore has the potential to monitor how environmental processes can influence population demography through monitoring development and fecundity over time. However, it will require much development to apply such methods to noninvasively collected samples. Innovations in sequencing platforms that do not require bisulphite conversion to examine methylation patterns, such as PacBio (Rhoads & Au, 2015), will be useful. Studies that evaluate how the DNA degradation that often occurs in noninvasive genetic sampling impacts assay methods will also be required.

#### 4.2.2 Monitoring health

The microbial communities living on or in multicellular organisms or “hosts,” termed microbiomes, are a rich area of study in humans and, increasingly, wild animals. Host health and fitness can be affected by the microbiome through different mechanisms: the microbiome could act directly to protect health, through competitive exclusion or by stimulating immunity, or act indirectly, by modifying metabolism or development (Bahrndorff, Alemu, Alemneh, & Lund Nielsen, 2016). For example, research has linked changes in skin bacterial microbiome with outbreaks of chytrid fungus in endangered frog populations (e.g., Jani & Briggs, 2014), and there is evidence that symbiotic bacteria on amphibian skin generate metabolites protective against the fungus (Loudon et al., 2014). Additionally, the microbiome might include known pathogens (Acevedo‐Whitehouse, Rocha‐Gosselin, & Gendron, 2010; Delgado et al., 2017): long‐term, noninvasive monitoring of the of the southern resident killer whale population in North America showed that antibiotic‐resistant bacteria were present in the respiratory microbiome of apparently healthy individuals (Raverty et al., 2017). Therefore, microbiomes could be regularly screened using MIS for the presence of both beneficial and harmful components as part of an ongoing genetic monitoring scheme. Changes in the characteristics of the microbiome over time might also be indicative of changes in the quality of the social or broader environment (Amato et al., 2013; Tung et al., 2015) and can be significantly differentiated among individuals within a population (Klein‐Jöbstl et al., 2014). Additionally, studies in model organisms have used proteomic analysis of faecal samples to noninvasively monitor host–microbe interaction during development (e.g., Young et al., 2015) and disease processes (e.g., Yau, Leong, Zeng, & Wasinger, 2013).

As the gut microbiome is closely related to diet (Amato et al., 2013; Delsuc et al., 2014), it has been suggested as a potential screening tool to identify dietary components (Bahrndorff et al., 2016). However, evidence suggests that survey methods focusing on noninvasively collected faecal samples need to carefully consider the change in microbiome linked to environmental conditions, time since deposition and focal species (Menke, Meier, & Sommer, 2015).

#### 4.2.3 Functional or adaptive genetic monitoring

Traditional genetic monitoring has focused on presumably neutral markers to identify individuals and to assess genetic diversity. When whole‐genome data are available, investigators have the choice of using intergenic SNPs from gene deserts or of using “non‐neutral” markers derived from protein‐coding genes thought to be targets of natural selection (DeWoody et al., 2017; Doyle et al., 2016). This can be an important distinction, because the non‐neutral loci are often more sensitive indicators of population differentiation (Freamo, O'reilly, Berg, Lien, & Boulding, 2011). By combining genomic and environmental data, landscape genomics approaches can also be a powerful approach to infer and define conservation units (Funk, McKay, Hohenlohe, & Allendorf, 2012).

Only a few studies have yet investigated the possibilities of using MIS approaches for such a purpose. Russello et al. (2015) used hair samples to investigate genetic diversity and in the American pika hair, detecting several candidate gene regions which exhibited putative signatures of divergent selection for adaptation to altitude. Given the potential environment shifts related to climate change that can be expected, landscape genomics may offer useful insight to better monitor and manage wild and domestic populations.

## 5 CONCLUSION

Genetic monitoring with MIS has proven to be a valuable tool to monitor and manage species and populations. With increasing access to new technological advances, researchers will be able to go beyond identifying individuals to investigate their role in the ecosystem and assess population‐level dynamics. Such tools will be necessary to meet the challenges of conservation biology in a rapidly changing environment.

## DISCLAIMER

The views expressed in this information product are those of the authors and do not necessarily reflect the views or policies of FAO.

## CONFLICT OF INTEREST

The author declares no conflict of interest.
